# Clinical and Microbiological Efficacy of Adjunctive Systemic Quinolones to Mechanical Therapy in Periodontitis: A Systematic Review of the Literature

**DOI:** 10.1155/2022/4334269

**Published:** 2022-05-21

**Authors:** Carlos-M. Ardila, Jader-Alexander Bedoya-García

**Affiliations:** ^1^Universidad de Antioquia U de A, Medellín, Colombia; ^2^Biomedical Stomatology Research Group, Universidad de Antioquia U de A, Medellín, Colombia

## Abstract

**Objectives:**

To assess the clinical and microbiological efficacy of systemic quinolones adjunctive to mechanical therapy in periodontitis patients*. Materials and Methods*. *A* systematic review of the scientific literature was carried out. The search scheme comprised the Scopus, PubMed/MEDLINE, SCIELO (Scientific Electronic Library Online), and LILACS (Literatura Latinoamericana *y* del Caribe en Ciencias de la Salud) databases, together with the gray literature. MeSH terms and keywords were utilized to explore publications in all idioms. Only randomized clinical trials (RCTs) that met the selection criteria were included.

**Results:**

A total of 4 RCTs were selected. These RCTs found superior clinical and microbiological efficacy of adjunctive systemic moxifloxacin (MOX) and levofloxacin (LV) compared to subgingival debridement plus placebo. Improvements in PD and CAL were 2.4 ± 0.8 mm and 2.7 ± 0.9 mm for LV, and 1.5 ± 0.5 mm and 1.8 ± 0.5 mm for MOX, respectively. After six months of follow-up, adjunctive MOX reduced the presence of *Aggregatibacter actinomycetemcomitans* to imperceptible levels, while LV markedly reduced this microorganism. Some adverse events were reported in the LV group and none in the MOX group.

**Conclusions:**

Adjunctive MOX and LV improve probing depth and clinical attachment level compared with subgingival debridement alone in patients with periodontitis. The efficacy of these quinolones against *A. actinomycetemcomitans* was also superior.

## 1. Introduction

Periodontitis is one of the most common inflammatory diseases in the world [1]. In 2019, there were 1.1 billion prevalent cases of severe periodontitis estimated globally [2]. Consequently, improving actions for prompt diagnosis and therapy of periodontitis can be relevant to constraining the systemic harm that periodontitis can exert [3]. Thus, it has been shown that periodontitis is a significant predictor of serum Galectin-3 levels and nod-like receptor family pyrin domain-containing protein-3, biomarkers of endothelial dysfunction in patients with periodontitis, coronary heart disease, and type-II diabetes mellitus [3, 4].

It is widely recognized that the treatment of periodontitis involves nonsurgical mechanical removal of bacterial biofilm related to inflammation of all supragingival and subgingival surfaces, in addition to regular hygiene performed by the patient, accompanied by professional maintenance [5].

Different systematic reviews have shown an additional benefit when adjunctive antimicrobials are used [1, 6–8]. However, a consensus has suggested that these additional benefits are of clinical importance in patients with severe periodontitis aged 55 years and younger [1]. Moreover, scientific evidence has revealed that in these cases, the best management for treating periodontitis is mechanical therapy (subgingival debridement) plus amoxicillin/metronidazole (A + M) ( [1, 6–9]. Nevertheless, it is fundamental to accentuate that A + M must be recommended with care because several periodontopathogens have shown antimicrobial resistance in diverse countries [10–13]. Contemplating this aspect and the high occurrence of hypersensitivity reactions to beta-lactam antimicrobials, quinolones can represent a pharmacological prospect [14]. Furthermore, the characteristics of some quinolones, such as moxifloxacin (MOX) and levofloxacin (LV), allow the administration of a single dose per day, reducing costs and increasing patient satisfaction [15, 16]. This is a very important aspect because partial compliance with the adjunctive antimicrobial protocol intake reduces the efficacy of periodontal therapy [17].

Different systematic reviews have also indicated the importance of evaluating the permanence of the results of clinical trials with adjuvants for the treatment of periodontitis during a follow-up period of at least 6 months [1, 6–8].

In this context, it is relevant to carry out a systematic review of clinical trials with a follow-up of at least 6 months, which allows for evaluating the clinical and microbiological efficacy of adjunct systemic quinolones in the treatment of periodontitis.

## 2. Materials and Methods

### 2.1. Protocol Registration

This review of clinical trials was carried out considering the PRISMA (Preferred Reporting Items for Systematic Reviews and Meta-analyses) guide [18]. Moreover, the protocol of this study was registered in PROSPERO (International Prospective Register of Systematic Reviews-receipt 3189651).

### 2.2. Study Eligibility and Selection Criteria

The PICOS question was formulated as follows.

The population included patients with periodontitis, nonsmokers, and those with no systemic diseases.

The intervention was subgingival debridement (scaling and root planing) plus an adjunctive systemic quinolone.

### 2.3. Comparison of Subgingival Debridement

The primary outcome included improvement of clinical parameters in terms of probing depth (PD) and clinical attachment level (CAL). Secondary outcomes such as bleeding on probing (BOP), decreased presence of microorganisms, and reporting of adverse events were also considered.

The study design was randomized clinical trials (RCTs) with at least 6 months of follow-up.

### 2.4. Search Strategy

The search scheme comprised the SCOPUS, PubMed/MEDLINE, SCIELO, and LILACS databases, together with the gray literature (OpenGrey and Google Scholar). Keywords and MeSH terms were utilized to explore publications in all idioms until February 2022, incorporating the terminologies periodontitis, periodontal diseases, aggressive periodontitis, chronic periodontitis, rapidly progressive periodontitis, early-onset periodontitis, adult periodontitis, quinolones, moxifloxacin, ciprofloxacin, levofloxacin, periodontal treatment, subgingival debridement, scaling and root planing, mechanical therapy, adjunctive antimicrobials, and RCTs issued in all idioms. The next exploration procedure was utilized to research databases, using Boolean operators (AND, OR): “periodontitis/subgingival debridement” OR “periodontitis/scaling and root planing” OR “periodontitis/mechanical therapy” OR “periodontitis/adjunctive antimicrobials” OR “periodontal pocket/treatment” OR “clinical attachment loss/treatment” AND “adjunctive antimicrobials” OR “quinolones” OR “ciprofloxacin” OR ““moxifloxacin” OR “levofloxacin.”

Only RCTs that incorporated at least 6 months of follow-up were elected, involving systematically healthy persons diagnosed with periodontitis and managed with systemic quinolones adjunctive to subgingival debridement. RCTs that managed patients surgically, duplicate publications, RCTs that utilized antimicrobials in subantimicrobial prescriptions, in vitro experimentations, and investigations implemented on animals were discarded.

### 2.5. Review Process

The two investigators reviewed the titles and abstracts and selected RCTs to assess the full text for potential eligibility. In case of disagreement between authors, trial eligibility was made by consensus. The Kappa statistical test was used to assess the value of agreement between observers (>95).

### 2.6. Data Collection

A table was designed to incorporate the most relevant data from the selected RCTs. This process was performed independently by each of the researchers. Subsequently, the data were compared. Recorded data included authors' names, date of publication, periodontal diagnosis, age and gender of participants, intervention, control, number of participants, comparison between the groups (main outcome variables), and length of the follow-up period.

### 2.7. Risk of Bias

The risk of bias and quality assessment of the included trials was performed following the Jadad scale [19] for RCTs, by the two authors.

## 3. Results

**Figure 1:**
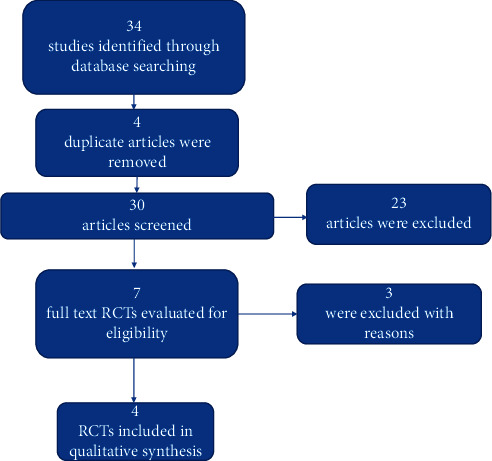
Flowchart of the RCTs selection method.

The electronic search yielded 34 studies. After reviewing the titles and abstracts, 23 investigations were excluded for their irrelevance and 4 duplicate articles were also removed. Reading the full text resulted in the exclusion of 3 additional trials because they did not use a control group with subgingival debridement plus a placebo. Finally, only 4 RCTs were included in this systematic review (Figure 1).

The characteristics of the included studies are presented in Table 1. All RCTs were double-blind and placebo-controlled, with parallel design. These RCTs were published between 2014 and 2020.

These experiments evaluated 210 patients with a minimum sample of 36 patients [22] and a maximum of 69 [20]. One RCT included patients diagnosed with generalized severe chronic periodontitis [16], two with generalized aggressive periodontitis [21, 22], and one experiment with severe periodontitis [20]. In all RCTs, full-mouth subgingival debridement was performed in a single session. Quinolones adjunct to a mechanical therapy included MOX in two trials [21, 22] and LV in two RCTs [16, 20]. One trial included three arms; in addition to MOX and the control group, an A + M group was also studied [22]. All the studies had a follow-up of 6 months. All the studies evaluated clinical (PD, CAL, and BOP) and microbiological parameters.

All RCTs showed a statistically significant greater reduction in PD and improvement in CAL compared to the control group. The range of preoperative measurements for the experiments involving LV was 6.1–6.6 mm for PD and 6.6–7.8 mm for CAL; while for MOX, they were 4.2–4.8 mm and 4.8–4.9 mm, respectively. On the other hand, the range of postoperative measurements for LV was 3.82–4.04 mm (PD) and 4.06–5.05 mm (CAL), and for MOX, they were 3.02–3.08 mm (PD) and 3.05–3.14 mm (CAL). Therefore, the improvements in PD and CAL were 2.4 ± 0.8 mm and 2.7 ± 0.9 mm for LV and 1.5 ± 0.5 mm and 1.8 ± 0.5 mm for MOX, respectively. No differences in BOP were observed between groups in any RCT. The RCT by Ardila et al. [22] that also compared MOX with A + M showed no statistically significant differences between the clinical parameters.

In all RCTs, the microbiological analysis was accomplished in all patients at baseline, 3 months, and 6 months after treatment, and the samples were collected from the deepest site of each quadrant. For the identification of microorganisms, two RCTs used polymerase chain reaction [16, 22], while the other two used culture techniques [20, 21].

In all RCTs, the groups with adjunct antimicrobials showed a greater reduction in the occurrence of the periodontal pathogens studied. However, in the trial of Pradeep et al. [16], the differences between the percentage of positive patients in the test and control group were not significant at any time interval for *Porphyromonas gingivalis* and *Tannerella forsythia.* Interestingly, MOX reduced *A. actinomycetemcomitans* to undetectable levels [21, 22], while LV markedly reduced this microorganism [16, 20]. MOX and A + M showed a similar reduction in the occurrence of *P. gingivalis* and *T. forsythia* after 3 and 6 months, but MOX completely reduced the levels of *A.actinomycetemcomitans* [22].

The clinical trials that studied MOX did not report any adverse events. Only some patients in the A + M group reported nausea, diarrhea, and vomiting [22]. Some participants also reported dizziness and diarrhea in the trials that evaluated adjunctive LV [16, 20].

All the studies evaluated in this systematic review presented a low risk of bias (Table 2). However, the experiments included in this review presented great heterogeneity in their designs, as reflected in the use of different classes of antibiotics, dosage, different periodontal diagnoses, great variability in the characteristics of the patients studied, and variability in the microbiological analysis and the microorganisms studied, among other characteristics. Meta-analysis was not considered feasible.

## 4. Discussion

To the best of the authors' understanding, this systematic review is the first to assess the clinical and microbiological efficacy of adjunct systemic quinolones in the treatment of periodontitis with at least 6 months of follow-up. Previously, a systematic review evaluated the therapeutic benefits of fluoroquinolones [23]. However, it has a highly questionable level of evidence because it included studies with some elements that generate many biases; for example, smoking patients, antimicrobial subdoses, quinolones adjunct to surgical therapy, and very short follow-up time, among others.

The strict selection criteria of this systematic review allowed to include only 4 RCTs, two with adjunctive MOX [21, 22] and two with adjunctive LV [16, 20].

MOX is a fourth-generation quinolone with good tolerability and bioavailability, a prolonged half-life, and satisfactory tissue distribution [24]. LV is the synthetic L-isomer of the racemic quinolone ofloxacin. It is quickly absorbed and dispersed broadly in tissues and fluids [25]. MOX has applied valuable antimicrobial results against periodontopathogens and Gram-negative enteric rods [15, 26]. LV is also effective against an extensive variety of Gram-positive, Gram-negative, and atypical microorganisms [27]. Moreover, fluoroquinolones are recognized to provoke an immunological reaction, permitting the elimination of *A. actinomycetemcomitans* [28]. However, negative effects of fluoroquinolones in young people associated with musculoskeletal concerns have been documented, but they emerge more regularly with LV. Thus, the use of LV has not been recommended in patients under 18 years of age [29].

This review found improvement in PD and CAL levels. These results corroborate those reported by other systematic reviews that indicate that the adjunctive management of systemic antimicrobials in the active phase of periodontitis therapy led to a statistically significant supplementary full-mouth PD diminution and CAL benefit [7, 30]. The results of the review by Teughels et al. [30] also highlight the benefits of MOX in these parameters. A similar situation is documented by Khattri et al. [7] during adjunctive systemic use of MOX and LV in periodontitis treatment.

The results of this review do not show statistically significant differences in BOP levels when comparing experimental and control groups during follow-up. Similar results were described by a recent review [6].

It was observed that systemically adjunctive MOX presented high microbiological efficacy against *P. gingivalis* and *T. forsythia* and *A. actinomycetemcomitans* was decreased to imperceptible levels [21, 22]. Other in vitro studies [26, 28] and clinical trials [31] corroborate these results. On the other hand, adjunctive systemic LV showed a reduction in *P. gingivalis* and *T. forsythia* levels but without statistically significant differences compared to the control group [16]. However, the decrease in *A. actinomycetemcomitans* was significant [20]. Therefore, these effects make the administration of these adjunctive systemic quinolones recommendable in the therapy of periodontitis patients harboring *A. actinomycetemcomitans*.

The use of LV reported adverse events such as dizziness, nausea, and vomiting in the two trials included in this review [16, 20]. In contrast, no adverse events were reported in the two RCTs that studied MOX [21, 22]. Similarly, the clinical trial by Guentsch et al. [31] did not describe any adverse events during MOX administration in patients with periodontitis.

The clinical trials evaluated in this systematic review were characterized by their quality and high level of evidence aspects that were also highlighted by other authors in different systematic reviews [6, 7, 30]. However, considering that the experiments included in this review presented great heterogeneity, a meta-analysis was not considered feasible. The meta-analysis carried out by Romo et al. [23] which includes three of the four clinical trials selected in this review, corroborates this heterogeneity. The same authors indicate that due to this situation, their results should not be taken into consideration, as in turn recommended by Deeks and collaborators [32].

The main limitation of this systematic review is related to the small number of selected trials, an issue that was due to the rigorous selection criteria that allowed us to review studies with excellent evidence and low risk of bias. However, a greater number of studies with adequate follow-up periods are required to allow more conclusive results.

## 5. Conclusions

Adjunctive MOX and LV improve probing depth and clinical attachment level compared with subgingival debridement alone in patients with periodontitis. The efficacy of these quinolones against *A. actinomycetemcomitans* was also superior. Adjunctive systemic use of MOX revealed no adverse events. Consequently, MOX and LV can be considered therapeutic options for the treatment of periodontitis.

## Figures and Tables

**Table 1:** Features of the RCTs evaluated.

Authors/publication date	Periodontal diagnoses	Participants	Mean age	Female/male	Intervention/control	Main outcomes	Follow-up
[20]	Severe periodontitis	66	37 years	32/34	LV 500 mg OD x 10 days SD + placebo	Mean PD and CAL were improved after 3 and 6 months with statistically significant differences compared to the control group. No differences were observed in BOP. The presence of *Aa* was significantly lower in the experimental group.	6 months

[21]	Aggressive periodontitis	40	27 years	23/17	MOX 400 mg OD x 7 days SD+placebo	Mean PD and CAL were improved after 3 and 6 months with statistically significant differences compared to the control group. No differences were observed in BOP. MOX group showed a significantly superior decrease in the occurrence of patients colonized by all the periodontal pathogens studied, at 3 months and 6 months. *Aa* was reduced to undetectable levels in the MOX group.	6 months

[16]	Chronic periodontitis	65	37 years	34/31	LV 500 mg OD x 10 days SD + placebo	Mean PD and CAL were improved after 3 and 6 months with statistically significant differences compared to the control group. No differences were observed in BOP. The presence of *Aa* was significantly lower in the experimental group. The differences between the percentage of positive patients in the test and control groups were not significant at any time interval for *Pg* and *Tf.*	

[22]	Aggressive periodontitis	36	26 years	23/13	MOX 400 mg OD x 7 days A+M 500 mg TID x 7 day SD+placebo	The antimicrobial groups showed statistically significant greater improvement in PD and CAL after 3 and 6 months compared to the control group No differences were observed in BOP. The antimicrobial groups presented a significantly higher reduction in the levels of periodontopathogens. *Aa* was reduced to undetectable levels in the MOX group.	6 months

PD = probing depth; CAL = clinical attachment level; BOP=bleeding on probing; MOX = moxifloxacin; LV = levofloxacin; SD = subgingival debridement; OD = once a day; TID = three times a day; Aa = *Aggregatibacter actinomycetemcomitans*; Pg = *Porphyromonas gingivalis*; Tf = *Tannerella forsythia*.

**Table 2:** Quality of the selected studies [19].

RCT	Randomization	Blinding	Withdraw	Proper randomization	Proper blinding	Score
[20]	1	1	1	1	1	5
[21]	1	1	1	1	1	5
[16]	1	1	1	1	1	5
[22]	1	1	1	1	1	5

## Data Availability

Records were obtained from the included investigations.
